# Supplementary material for: Meta-analysis of reward processing in major depressive disorder reveals distinct abnormalities within the reward circuit
Source: Transl Psychiatry. 2019 Nov 11;9:293. doi: 10.1038/s41398-019-0644-x (PMC6848107; doi:10.1038/s41398-019-0644-x)
Supplement: Supplementary file 1 — Supplemental Material [file 41398_2019_644_MOESM1_ESM.docx]

**Meta-analysis of Reward Processing in Major Depressive Disorder Reveals
Distinct Abnormalities within the Reward Circuit**

***Supplementary Information***

**Supplementary Table 1**

*Comparison of Findings on Reward Responses (i.e., Reward > Punishment/Neutral) in Previous Meta-analyses*

| Brain Region | MNI Coordinates | | |
| --- | --- | --- | --- |
|  | x | y | z |
| *Groenewold et al.* ^19^ | | | |
| *MDD > HC* |  |  |  |
| Lingual Gyrus | 26 | -92 | -14 |
| Olfactorius Cortex | 4 | 22 | -14 |
| Middle Orbitofrontal | 2 | 26 | -14 |
| Rectus | 2 | 30 | -24 |
| Middle Orbitofrontal | 0 | 26 | -12 |
| Rectus | 0 | 24 | -24 |
| *HC > MDD* |  |  |  |
| Cerebellum | -16 | -74 | -28 |
| Lingual Gyrus | -18 | -62 | -6 |
| Fusiform Gyrus | -22 | -74 | -14 |
| Inferior Occipital Gyrus | -30 | -80 | -12 |
| Rolandic Operculum | -40 | -24 | 20 |
| Insula | -36 | -24 | 22 |
| Superior Temporal Gyrus | -40 | -36 | 12 |
| Heschl Gyrus | -46 | -16 | 12 |
| Postcentral Gyrus | -50 | -18 | 18 |
| Supramarginal Gyrus | -50 | -22 | 18 |
| Anterior Cingulate Cortex | -2 | 28 | 16 |
| Anterior Cingulate Cortex | 4 | 32 | 14 |
| Lingual Gyrus | -18 | -62 | -6 |
| Cerebellum | -6 | -58 | -4 |
| Calcarine Sulcus | -20 | -54 | 4 |
| Fusiform Gyrus | -26 | -58 | -12 |
| Precuneus | -20 | -52 | 2 |
| Pallidum | 18 | 0 | -4 |
| Putamen | 28 | -4 | 8 |
| Thalamus | 14 | -8 | 0 |
| Insula | 38 | 10 | -12 |
| Amygdala | 30 | -2 | -12 |
| Caudate | 16 | 26 | 6 |
| Fusiform | 44 | -62 | -20 |
| Crus Cerebellum | 44 | -64 | -20 |
| Brain Region | TAL Coordinates | | |
|  | x | y | z |
| *Zhang et al.* ^20^ | | | |
| *MDD > HC* |  |  |  |
| Cuneus | 4 | -86 | 18 |
| Cuneus | -6 | -86 | 22 |
| Frontal Lobe | 20 | 30 | -6 |
| Middle Frontal Gyrus | 40 | 28 | 38 |
| Superior Frontal Gyrus | -4 | 48 | 32 |
| Fusiform Gyrus | -48 | -74 | -12 |
| Middle Frontal Gyrus | -48 | 14 | 30 |
| Lingual Gyrus | 12 | -52 | 4 |
| Lingual Gyrus | 14 | -54 | 0 |
| *HC > MDD* |  |  |  |
| Caudate | -6 | 18 | 4 |
| Caudate | -8 | -8 | 10 |
| Thalamus | -10 | -12 | 8 |
| Thalamus | -14 | -14 | 16 |
| Caudate | -12 | -4 | 20 |
| Cerebellum | 4 | -36 | -4 |
| Cerebellum | -4 | -42 | 4 |
| Putamen | 14 | 8 | 2 |
| Caudate | 14 | 14 | 10 |
| Anterior Cingulate | -8 | 30 | 10 |
| Insula | 34 | -4 | 16 |
| Cerebellum | -6 | -60 | -20 |
| Brain Region | Coordinates | | |
|  | x | y | z |
| *Keren et al.* ^21^ | | | |
| *HC > MDD* |  |  |  |
| Caudate Body | 12 | 14 | 14 |
| Caudate Head | 6 | 2 | -2 |
| Caudate Body | -8 | -2 | -18 |

Abbreviations: MNI, Montreal Neurological Institute space; MDD, major depressive disorder; HC, healthy controls; TAL, Talairach space. Ventral striatum is the only area implicated in reward processing in MDD relative to HCs across the two previous meta-analyses and the current meta-analysis (see Table 1 for peak coordinates of group differences in neural responses to reward found in the current meta-analysis).

**Supplementary Table 2**

*Characteristics of the Study Samples Included in the Meta-Analysis.*

|  |  | MDD Patients | | | | |  |  | Healthy Controls | | |
| --- | --- | --- | --- | --- | --- | --- | --- | --- | --- | --- | --- |
| Study | Diagnostic Criteria | *n* | Age | % Female | % Medicated | Mood States | Comorbidity |  | *n* | Age | % Female |
| Arrondo *et al.* ^22^ | DSM-IV | 24 | 33.1 | 29.2% | 54.2% | D | Exclusion of alcohol or drug dependence. |  | 21 | 34.3 | 23.5% |
| Bremner *et al.* ^23^ | DSM-IV | 18 | 40 | 66.7% | 0.0% | D | Exclusion of organic mental disorders or comorbid psychotic disorders, post-traumatic stress disorder, childhood trauma, alcohol or substance abuse or dependence, or dyslexia. No current or past history of comorbid psychiatric disorders. |  | 9 | 35 | 77.8% |
| Burger *et al.* ^24^ | DSM-IV | 36 | 40.7 | 61.1% | 100.0% | D | Exclusion of substance dependence. Inclusion of PD, agoraphobia, generalized anxiety disorder, social phobia, obsessive compulsive disorder, post-traumatic stress disorder, somatoform disorder, eating disorder, dysthymia, alcohol abuse, and substance abuse. |  | 36 | 41.3 | 52.8% |
| Chantiluke *et al.* ^25^ | DSM-IV | 20 | 16.2 | 50.0% | 0.0% | D | Exclusion of major psychiatric disorders. |  | 21 | 16.3 | 52.4% |
| Chase *et al.* ^26^ | DSM-IV | 40 | 31 | 77.5% | 77.5% | D | No exclusion of psychiatric comorbidities. Inclusion of lifetime comorbid anxiety disorders and substance use disorders. |  | 37 | 33.1 | 67.6% |
| Demenescu *et al.* ^1^ | DSM-IV | 59 | 36.2 | 66.1% | 23.7% | D | Exclusion of axis I disorders, such as psychotic disorder or dementia, current alcohol or substance abuse. |  | 56 | 39.8 | 60.7% |
| Dichter *et al.* ^27^ | DSM-IV | 19 | 23.6 | 78.9% | 0.0% | R | Exclusion of current axis I psychopathology. |  | 19 | 27.9 | 63.2% |
| Elliott *et al.* ^28^ | DSM-IV | 10 | 42.2 | 70.0% | 100.0% | D | Exclusion of current comorbid anxiety disorders, substance abuse or dependence, bipolar disorder, or other psychiatric diagnoses. Inclusion of past history of PD and bulimia. |  | 11 | 37.6 | 72.7% |
| Engelmann *et al*. ^29^ | DSM-IV | 19 | 37.6 | 52.6% | 0.0% | D | Exclusion of lifetime bipolar disorder, psychotic disorder, obsessive-compulsive disorder, tic disorder, eating disorder, cognitive disorder, substance abuse or dependence in the previous 6 months or positive urine drug screen, or clinically significant suicidal ideation. |  | 23 | 33.7 | 60.9% |
| Fournier *et al.* ^30^ | DSM-IV | 26 | 30.6 | 69.0% | 69.2% | D | Exclusion of bipolar disorder, borderline personality disorder, and alcohol/substance use disorder within 2 months before the scan. Inclusion of history of anxiety disorder and substance abuse. |  | 28 | 32.6 | 57.0% |
| Fu *et al.* ^31^ and ^32^ | DSM-IV | 19 | 43.2 | 68.4% | 100.0% | D | Exclusion of current axis I disorder and history of substance abuse within 2 months of study participation. |  | 19 | 42.8 | 57.9% |
| Fu *et al.* ^33^ | DSM-IV | 16 | 40 | 81.3% | 0.0% | D | Exclusion of other axis I disorder, including anxiety disorder or history of substance within 2 months of study participation. |  | 16 | 39.2 | 81.3% |
| Gotlib *et al.* ^34^ | DSM-IV | 18 | 35.2 | 72.2% | 50.0% | D | Exclusion of psychotic ideation, social phobia, PD, mania, or substance abuse in the past 6 months or behavioral indications of possible impaired mental status. |  | 18 | 30.8 | 72.2% |
| Gradin *et al.* ^35^ | DSM-IV | 25 | 25.5 | 68.0% | 0.0% | D | Unspecified |  | 25 | 25.4 | 68.0% |
| Hall *et al.* ^36^ | DSM-IV | 29 | 37.4 | 55.2% | 51.7% | D | Exclusion of history of alcohol or substance abuse. |  | 25 | 37.7 | 55.2% |
| Johnston *et al.* ^37^ | DSM-IV/ ICD-10 | 19 | 50.8 | 78.9% | 85.0% | D | Exclusion of other primary psychiatric disorder and substance misuse. |  | 21 | 46.1 | 71.4% |
| Keedwell *et al.* ^38^ | ICD-10 | 12 | 43 | 66.7% | 66.7% | D | Exclusion of other axis I disorder. |  | 12 | 36 | 66.7% |
| Knutson *et al.* ^39^ | DSM-III-R | 14 | 30.7 | 64.3% | 0.0% | D | Exclusion of other current axis I disorder. |  | 12 | 28.7 | 66.7% |
| Kumari *et al.* ^40^ | DSM-IV | 6 | 47 | 100.0% | Unspecified | D | Unspecified |  | 6 | 44 | 100.0% |
| Laurent *et al.* ^41^ | DSM-IV | 11 | 24.1 (whole sample) | 100.0% | 23.1% | D | No exclusion of psychiatric comorbidities. Inclusion of past substance abuse/dependence, anxiety disorders, and eating disorder. |  | 11 | 24.1 (whole sample) | 100.0% |
| Liu *et al*. ^42^ | DSM-IV | 21 | 30.7 | 57.1% | 0.0% | D | Exclusion of axis I disorders (other than anxiety) and psychotic features and lifetime substance abuse or dependence. |  | 17 | 28.3 | 58.8% |
| Murrough *et al.* ^43^ | DSM-IV | 20 | 38.1 | 44.4% | 0.0% | D | Exclusion of lifetime history of psychotic illness or bipolar disorder and current alcohol or substance abuse. |  | 20 | 35 | 45.0% |
| Pizzagalli *et al.* ^44^ | DSM-IV | 30 | 43.2 | 50.0% | 0.0% | D | Exclusion of other axis I disorder except for anxiety disorders. |  | 31 | 38.8 | 41.9% |
| Remijnse *et al.* ^45^ | DSM-IV | 20 | 35 | 40.0% | 0.0% | D | Exclusion of current alcohol or substance abuse at the time of study participation. Inclusion of social anxiety disorder, generalized anxiety disorder, PD without agoraphobia, PD, and cannabis abuse in early and sustained full remission. |  | 27 | 32 | 70.4% |
| Rizvi *et al.* ^46^ | DSM-IV | 21 | 38.9 | 66.7% | 0.0% | D | Exclusion of other primary axis I disorder, lifetime history of hypomania/mania, psychosis, obsessive compulsive disorder, or eating disorder, and substance abuse or dependence (except nicotine or caffeine) within the last 3 months. |  | 18 | 36.2 | 66.7% |
| Rosenblau *et al.* ^47^ | DSM-IV | 12 | 43.5 | 41.7% | 0.0% | D | Exclusion of other axis I or II disorders. |  | 12 | 45.8 | 41.7% |
| Scheuerecker *et al.* ^48^ | DSM-IV | 13 | 37.9 | 23.1% | 0.0% | D | Exclusion of past alcohol or substance abuse, other mental illnesses, and personality disorders. |  | 15 | 35.5 | 33.3% |
| Schiller *et al.* ^49^ | DSM-IV | 19 | 23.6 | 78.9% | 0.0% | R | Exclusion of current axis I psychopathology. |  | 19 | 27.9 | 63.2% |
| Segarra *et al.* ^50^ | DSM-IV | 24 | 33.1 | 29.2% | 54.0% | D | Exclusion of dependence on alcohol or recreational drugs. |  | 21 | 34.3 | 19.0% |
| Sharp *et al.* ^51^ | DSM-IV | 14 | 13.4 | 100.0% | Unspecified | D | Exclusion of current use of nicotine, illicit drugs, psychotic disorders, bipolar I disorder, learning disabilities, and mental retardation. |  | 19 | 13.7 | 100.0% |
| Smoski *et al.* ^52^ | DSM-IV | 14 | 34.8 | 50.0% | 0.0% | D | Exclusion of current mood disorder, anxiety disorder, psychotic disorder, substance abuse, or active suicidal ideation and history of psychosis or mania. |  | 15 | 30.8 | 60.0% |
| Smoski *et al.* ^53^ | DSM-IV | 9 | 34.4 | Unspecified | 44.4% | D | Inclusion of generalized anxiety disorder and binge eating disorder. |  | 13 | 26.2 | Unspecified |
| Surguladze *et al.* ^54^ | DSM-IV | 16 | 42.3 | 37.5% | 100.0% | D | Exclusion of illicit substance abuse. |  | 14 | 35.1 | 42.9% |
| Surguladze *et al.* ^55^ | DSM-IV | 9 | 42.8 | 44.4% | 100.0% | D | Exclusion of illicit substance abuse and other axis I disorders. |  | 9 | 39.7 | 44.4% |
| Townsend *et al.* ^56^ | DSM-IV | 15 | 45.6 | 40.0% | 0.0% | D | Exclusion of comorbid axis I disorder. |  | 15 | 44.8 | 40.0% |
| Wagner *et al.* ^2^ | DSM-IV | 19 | 39.9 | 55.0% | 100.0% | D | Exclusion of current comorbid axis I disorder and a history of manic episodes. |  | 20 | 34.1 | 60.0% |
| Wang *et al.* ^57^ | DSM-IV | 12 | 69.1 | 58.3% | 91.7% | D | Exclusion of another major psychiatric disorder and alcohol/drug abuse/dependence. Inclusion of generalized anxiety disorder. |  | 20 | 73.1 | 60.0% |
| Young *et al.* ^58^ | DSM-IV-TR | 16 | 37.1 | 87.5% | 0.0% | D | Exclusion of serious suicidal ideation, psychosis, drug/alcohol abuse in the past year and dependence (except for nicotine) in their lifetime. |  | 16 | 37.8 | 87.5% |
| Zhang *et al.* ^59^ | ICD-10 | 21 | 43.8 | 38.1% | 100.0% | D | Exclusion of illicit substance use or substance use disorders. |  | 25 | 39.3 | 36.0% |
| Zhong *et al.* ^60^ | DSM-IV | 29 | 20.5 | 55.2% | 0.0% | D | Exclusion of lifetime substance dependence and substance abuse in the last 6 months. |  | 31 | 20.8 | 51.6% |

Abbreviations: MDD, major depressive disorder; D, depressed; R, remitted; PD, panic disorder.

**Supplementary Table 3**

*Characteristics of the Studies Included in the Meta-analysis*

| Study | fMRI or PET | Design | Space | Paradigm | Correction | Stimuli | Contrast |
| --- | --- | --- | --- | --- | --- | --- | --- |
| Arrondo *et al.* ^22^ | fMRI | Event-related | MNI | Modified monetary incentive delay task | Uncorrected | Money | HC > MDD, Anticipation: Reward > Non-Reward |
| Bremner *et al.* ^23^ | PET | Block | MNI | Verbal declarative memory tasks with neutral paragraph encoding compared to a control condition and sad word pair retrieval compared to a control condition. | Uncorrected at p < .005 | Words and paragraphs | MDD > HC, Outcome: Negative > Neutral  HC > MDD, Outcome: Negative > Neutral |
| Burger *et al.* ^24^ | fMRI | Event-related | MNI | Face matching paradigm | Corrected at p < .05 (TFCE) | Faces | HC > MDD, Outcome: Negative > Neutral  HC > MDD, Outcome: Positive > Neutral |
| Chantiluke *et al.* ^25^ | fMRI | Event-related | TAL | Reward continuous performance task | Uncorrected at p < .005 | Money | MDD > HC, Outcome: Reward > Non-Reward  HC > MDD, Outcome: Reward > Non-Reward |
| Chase *et al.* ^26^ | fMRI | Event-related | MNI | Card guessing paradigm | Voxel-wise corrected at p < .05 and cluster-wise corrected at p < .01 | Money | MDD > HC, Anticipation: Reward > Non-Reward  HC > MDD, Anticipation: Reward > Non-Reward  MDD > HC, Anticipation: Reward Expectancy  HC > MDD, Anticipation: Reward Expectancy  MDD > HC, Outcome: Prediction Error |
| Demenescu *et al.* ^1^ | fMRI | Event-related | MNI | Viewing faces with angry, fearful, sad, happy, and neutral expressions and scrambled faces; rating gender or pressing buttons in conformity with the instruction presented on the screen | Cluster-wise corrected at p < .05 | Faces | MDD > HC, Outcome: Positive > Scrambled Face |
| Dichter *et al.* ^27^ | fMRI | Event-related | MNI | Modified monetary incentive delay task | Uncorrected at p < .005, k ≥ 10 | Money | MDD > HC, Anticipation: Reward > Non-Reward  MDD > HC, Outcome: Reward > Non-Reward  HC > MDD, Outcome: Reward > Non-Reward |
| Elliott *et al.* ^28^ | fMRI | Block | MNI | Affective go/no go task | Uncorrected at p < .001 | Words | MDD > HC, Outcome: Negative > Positive  HC > MDD, Outcome: Positive > Negative |
| Engelmann et al. ^29^ | fMRI | Event-related | MNI | Economic decision-making task | Cluster-wise corrected at p < .05 | Money | MDD > HC, Outcome: Negative > Positive |
| Fournier *et al.* ^30^ | fMRI | Block | MNI | Labeling a color flash superimposed upon neutral faces that gradually morphed into angry, fearful, sad, or happy faces | Uncorrected at p < .001, k > 20 | Faces | MDD > HC, Outcome: Negative > Neutral MDD > HC, Outcome: Positive > Neutral |
| Fu *et al.* ^31^ and ^32^ | fMRI | Event-related | TAL | Indicating the sex of faces morphed to represent low, medium, and high intensities of sadness | Cluster-wise corrected at p < .005 | Faces | MDD > HC, Outcome: Negative (low, medium, and high intensity)  HC > MDD, Outcome: Positive (low, medium, and high intensity) |
| Fu *et al.* ^33^ | fMRI | Event-related | TAL | Indicating the sex of faces morphed to represent low, medium, and high intensities of sadness | Unspecified | Faces | MDD > HC, Outcome: Negative (low, medium, and high intensity)  HC > MDD, Outcome: Negative (low, medium, and high intensity) |
| Gotlib *et al.* ^34^ | fMRI | Block | MNI | Indicating the sex of faces that were fearful, angry, sad, happy, neutral, or scrambled | Uncorrected at p < .001, k > 5 | Faces | MDD > HC, Outcome: Negative > Neutral  HC > MDD, Outcome: Negative > Neutral  MDD > HC, Outcome: Positive > Neutral  HC > MDD, Outcome: Positive > Neutral |
| Gradin *et al.* ^35^ | fMRI | Event-related | MNI | Ultimatum game | Cluster-wise corrected at p < .05 | Money | HC > MDD, Outcome: Increasing fairness (decreasing inequality)  MDD > HC, Outcome: Increasing inequality (decreasing fairness) |
| Hall *et al.* ^36^ | fMRI | Event-related | TAL | Contingency reversal reward paradigm | Voxel-wise corrected at p < .05 | Money | HC > MDD, Outcome: Magnitude of Loss: Large Loss > Small Loss  HC > MDD, Outcome: Magnitude of Reward: Large Reward > Small Reward  MDD > HC, Outcome: Reward Acquisition > Punishment Reversal  HC > MDD, Outcome: Reward Acquisition > Punishment Reversal |
| Johnston *et al.* ^37^ | fMRI | Event-related | MNI | Modified Pessiglione task | Cluster-wise corrected at p < .01 | Voucher | MDD > HC, Outcome: Loss > Non-Loss  HC > MDD, Outcome: Loss > Non-Loss  MDD > HC, Outcome: Reward > Non-Reward  HC > MDD, Outcome: Reward > Non-Reward |
| Keedwell *et al.* ^38^ | fMRI | Block | TAL | Being exposed to happy, sad, or neutral autobiographical memory prompts and facial expressions | Cluster-wise corrected at p < .01 | Autobiographical memory and faces | MDD > HC, Outcome: Negative > Neutral  HC > MDD, Outcome: Negative > Neutral  MDD > HC, Outcome: Positive > Neutral  HC > MDD, Outcome: Positive > Neutral |
| Knutson *et al.* ^39^ | fMRI | Event-related | TAL | Monetary incentive delay task | Uncorrected at p < .05 | Money | MDD > HC, Anticipation: Reward > Non-Reward  HC > MDD, Anticipation: Reward > Non-Reward  HC > MDD, Outcome: Non-Loss > Loss  HC > MDD, Outcome: Reward > Non-Reward |
| Kumari *et al.* ^40^ | fMRI | Block | TAL | Viewing positive or negative pictures with a caption | Cluster-wise corrected at p < .005 | Pictures and captions | HC > MDD, Outcome: Negative > Neutral  MDD > HC, Outcome: Negative > Neutral  HC > MDD, Outcome: Positive > Neutral  MDD > HC, Outcome: Positive > Neutral  HC > MDD, Outcome: Positive > Negative  MDD > HC, Outcome: Positive > Negative |
| Laurent *et al.* ^41^ | fMRI | Event-related | MNI | Seeing own infant vs. other infant distress faces | Cluster-wise corrected at p < .05 | Faces | HC > MDD, Outcome: Very negative > Negative |
| Liu et al. ^42^ | fMRI | Event-related | MNI | Instrumental probabilistic reward- and punishment-based associative learning task | Cluster-wise corrected at p < .05 | Money | MDD > HC, Outcome: Negative > Neutral  MDD > HC, Outcome: Punishment Prediction Errors |
| Murrough *et al.* ^43^ | fMRI | Event-related | MNI | Rating emotional valence of happy, sad, or neutral faces | Cluster-wise corrected at p < .05 | Faces | HC > MDD, Outcome: 100% Positive > Neutral |
| Pizzagalli *et al.* ^44^ | fMRI | Event-related | MNI | Monetary incentive delay task | Uncorrected at p < .005 | Money | MDD > HC, Anticipation: Loss > Non-Loss  HC > MDD, Anticipation: Loss > Non-Loss  MDD > HC, Anticipation: Reward > Non-Reward  HC > MDD, Anticipation: Reward > Non-Reward  MDD > HC, Outcome: Loss > Non-Loss  HC > MDD, Outcome: Loss > Non-Loss  MDD > HC, Outcome: Reward > Non-Reward  HC > MDD, Outcome: Reward > Non-Reward |
| Remijnse *et al.* ^45^ | fMRI | Event-related | MNI | Reversal learning task | Uncorrected p < .001 | Points | MDD > HC, Outcome: Loss > Baseline  HC > MDD, Outcome: Loss > Baseline  MDD > HC, Outcome: Reward > Baseline |
| Rizvi *et al.* ^46^ | fMRI | Blocked | MNI | Viewing IAPS pictures that elicit positive, negative or neutral affective states | Cluster-wise corrected at p < .05 | Pictures | MDD > HC, Outcome: Positive > Neutral  MDD > HC, Outcome: Negative > Neutral |
| Rosenblau *et al.* ^47^ | fMRI | Event-related | MNI | Viewing IAPS pictures that elicit positive, negative or neutral affective states with and without cues indicating their emotional valence | Uncorrected at p < .05 or p < .005 | Pictures | MDD > HC, Anticipation: Negative > Neutral  MDD > HC, Outcome: Negative > Neutral |
| Scheuerecker *et al.* ^48^ | fMRI | Block | MNI | Face matching paradigm | Uncorrected at p < .001 | Faces | MDD > HC, Outcome: Negative > Neutral |
| Schiller *et al.* ^49^ | fMRI | Event-related | MNI | Monetary incentive delay task | Cluster-wise corrected at p < .05 | Money | HC > MDD, Anticipation: Loss > Non-Loss  HC > MDD, Outcome: Loss > Non-Loss |
| Segarra *et al.* ^50^ | fMRI | Event-related | MNI | Simulated slot-machine game | Cluster-wise corrected at p < .05 | Money | HC > MDD, Outcome: Unexpected Reward > Full Miss |
| Sharp *et al.* ^51^ | fMRI | Event-related | TAL | Card guessing paradigm | Uncorrected at p < .005 | Money | HC > MDD, Outcome: Reward > Non-Reward |
| Smoski *et al.* ^53^ | fMRI | Event-related | MNI | Modified monetary incentive delay task | Cluster-wise corrected | Money | MDD > HC, Anticipation: Money > Control  HC > MDD, Anticipation: Money > Control  MDD > HC, Outcome: Non-Win > Control  HC > MDD, Outcome: Non-Win > Control  MDD > HC, Outcome: Winning > Control  HC > MDD, Outcome: Winning > Control  MDD > HC, Selection: Money > Control  HC > MDD, Selection: Money > Control |
| Smoski *et al.* ^52^ | fMRI | Event-related | MNI | Wheel of fortune task | Uncorrected at p < .005, k ≥ 10 | Money | HC > MDD, Anticipation: Reward > Non-Reward  HC > MDD, Outcome: Reward > Non-Reward |
| Surguladze *et al.* ^55^ | fMRI | Event-related | TAL | Indicating the sex of neutral faces and faces morphed to represent mild and high intensities of fear and disgust | Cluster-wise corrected at p < .001 | Faces | HC > MDD, Outcome: Increasing intensities of happy faces  MDD > HC, Outcome: Increasing intensities of sad faces |
| Surguladze *et al.* ^54^ | fMRI | Event-related | TAL | Indicating the sex of neutral faces and faces morphed to represent mild and high intensities of sadness and happiness | Cluster-wise corrected at p < .001 | Faces | MDD > HC, Outcome: Differential response to 100% disgust  HC > MDD, Outcome: Differential response to 50% fear |
| Townsend *et al.* ^56^ | fMRI | Block | MNI | Face matching paradigm | Cluster-wise corrected at p < .05 | Faces | HC > MDD, Outcome: Negative > Neutral |
| Wagner *et al.* ^2^ | fMRI | Event-related | MNI | Self-referential processing task | Cluster-wise corrected at p < .05 | Statements | MDD > HC, Outcome: Neutral > Negative  MDD > HC, Outcome: Neutral > Positive |
| Wang *et al.* ^57^ | fMRI | Event-related | MNI | Emotional oddball task | Uncorrected at p < .001, k = 5 | Pictures | MDD > HC, Outcome: Negative > Neutral |
| Young *et al.* ^58^ | fMRI | Event-related | TAL | Autobiographical memory task | Cluster-wise corrected at p < .05, k > 30 | Words and autobiographical memories | HC > MDD, Outcome: Very Positive > Positive  HC > MDD, Outcome: Very Negative > Negative  MDD > HC, Outcome: Very Negative > Negative |
| Zhang *et al.* ^59^ | fMRI | Event-related | MNI | Viewing IAPS positive, neutral, and negative pictures with or without valence cues | Cluster-wise corrected at p < .05, k > 157 | Pictures | MDD > HC, Outcome: Reward > Non-Reward |
| Zhong *et al.* ^60^ | fMRI | Block | MNI | Face matching paradigm | Uncorrected at p < .005, k =8 | Faces | MDD > HC, Outcome: Negative > Neutral  HC > MDD, Outcome: Negative > Neutral |

Abbreviations: fMRI, functional magnetic resonance imaging; PET, positron emission tomography; MNI, Montreal Neurological Institute space; SVC, small volume correction; MDD, major depressive disorder; HC, healthy controls; TFCE, threshold-free cluster enhancement; TAL, Talairach space; VS, ventral striatum; dACC, dorsal anterior cingulate cortex; rACC, rostral anterior cingulate cortex; ACC, anterior cingulate cortex; mPFC, medial prefrontal cortex; mOFC, medial orbitofrontal cortex; IAPS, International Affective Picture System.

**Supplementary Table 4**

*Peak Coordinates of Group Differences in Neural Responses to Reward (Excluding Neutral Stimuli > Punishment)*

| Contrast | Cluster Size (mm^3^) | Probabilistic Anatomical Label | x | y | z |
| --- | --- | --- | --- | --- | --- |
| MDD > HC | 968 | Frontal Orbital Cortex (26%), Frontal Pole (13%) | 20 | 32 | -12 |
| HC > MDD | 1784 | Subcallosal Cortex (14%) | -2 | 8 | -4 |
|  |  | Caudate (32.1%),  Accumbens (11.1%) | 8 | 6 | -2 |

Abbreviations: MDD, major depressive disorder; HC, healthy controls. Coordinates are x,y,z values of the locations of the maximum activation likelihood estimation (ALE) values in MNI space. Probabilistic labels reflect the probability that a coordinate belongs to a given region derived from the Harvard-Oxford probabilistic atlas. For clarity, we only report labels whose likelihood exceeds 5%.


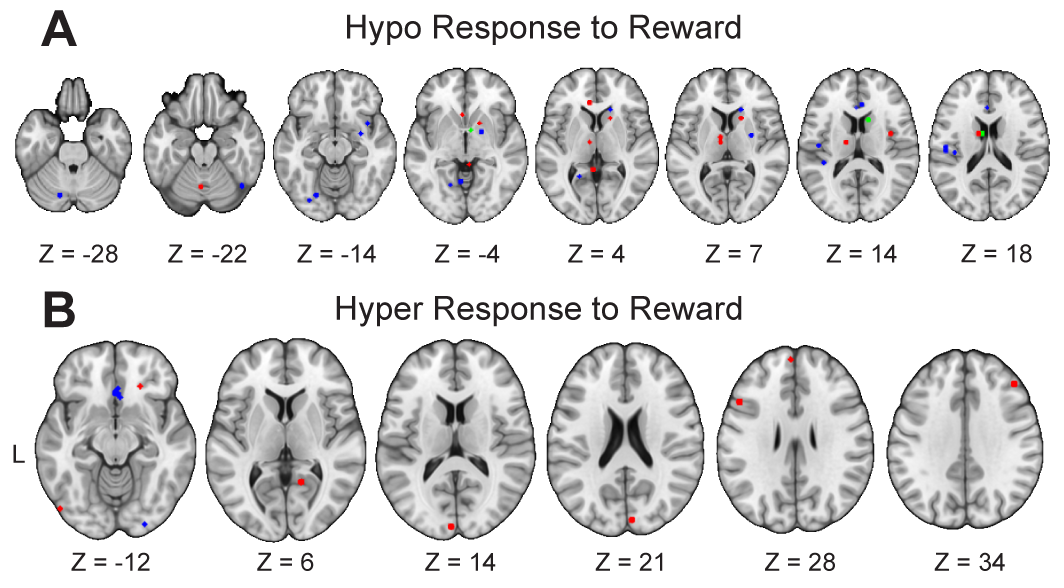


**Supplementary Figure 1**. Illustration of Findings of Previous Meta-analyses on Reward Processing in Unipolar Depression. There is a striking degree of anatomical disagreement across these meta-analyses, with non-overlapping findings all throughout the brain. Blue represents Groenewold et al.^19^ Green represents Keren et al.^21^ Red represents Zhang et al.^20^ **(A)** Previous meta-analyses examining convergence among studies reporting hypo-responses to reward include Groenewold et al.,^19^ Keren et al.,^21^ and Zhang et al.^20^ **(B)** Previous meta-analyses examining convergence among studies reporting hyper-responses to reward include Groenewold et al.^19^ and Zhang et al.^20^

**Supplementary Figure 2**. Hyper-responses to punishment in the sublenticular extended amygdala (SLEA) in major depressive disorder (MDD). To conduct exploratory analyses to examine which brain regions consistently show elevated response to punishment in MDD relative to healthy controls (HCs), we meta-analyzed 24 studies reporting greater activity in response to punishment in people with MDD than HCs. Our results indicated that these studies reliably report greater activation in the left SLEA in MDD.

**Supplementary References**

1 Demenescu LR, Renken R, Kortekaas R, van Tol M-J, Marsman JBC, van Buchem MA *et al.* Neural correlates of perception of emotional facial expressions in out-patients with mild-to-moderate depression and anxiety. A multicenter fMRI study. *Psychol Med* 2011; **41**: 2253–2264.

2 Wagner G, Schachtzabel C, Peikert G, Bär K-J. The neural basis of the abnormal self-referential processing and its impact on cognitive control in depressed patients. *Hum Brain Mapp* 2015; **36**: 2781–2794.

3 Roiser JP, Elliott R, Sahakian BJ. Cognitive Mechanisms of Treatment in Depression. *Neuropsychopharmacology* 2012; **37**: 117–136.

4 Eshel N, Roiser JP. Reward and Punishment Processing in Depression. *Biol Psychiatry* 2010; **68**: 118–124.

5 Hamilton JP, Etkin A, Furman DJ, Lemus MG, Johnson RF, Gotlib IH. Functional neuroimaging of major depressive disorder: A meta-analysis and new integration of base line activation and neural response data. *Am J Psychiatry* 2012; **169**: 693–703.

6 Price JL, Drevets WC. Neurocircuitry of Mood Disorders. *Neuropsychopharmacology* 2010; **35**: 192–216.

7 Rive MM, van Rooijen G, Veltman DJ, Phillips ML, Schene AH, Ruhé HG. Neural correlates of dysfunctional emotion regulation in major depressive disorder. A systematic review of neuroimaging studies. *Neurosci Biobehav Rev* 2013; **37**: 2529–2553.

8 Phillips ML, Drevets WC, Rauch SL, Lane R. Neurobiology of emotion perception II: Implications for major psychiatric disorders. *Biol Psychiatry* 2003; **54**: 515–528.

9 Swartz JR, Knodt AR, Radtke SR, Hariri AR. A Neural Biomarker of Psychological Vulnerability to Future Life Stress. *Neuron* 2015; **85**: 505–511.

10 Mattson WI, Hyde LW, Shaw DS, Forbes EE, Monk CS. Clinical neuroprediction: Amygdala reactivity predicts depressive symptoms 2 years later. *Soc Cogn Affect Neurosci* 2016; **11**: 892–898.

11 Müller VI, Cieslik EC, Serbanescu I, Laird AR, Fox PT, Eickhoff SB. Altered brain activity in unipolar depression revisited: Meta-analyses of neuroimaging studies. *JAMA Psychiatry* 2017; **74**: 47–55.

12 Ferenczi EA, Zalocusky KA, Liston C, Grosenick L, Warden MR, Amatya D *et al.* Prefrontal cortical regulation of brainwide circuit dynamics and reward-related behavior. *Science* 2016; **351**: aac9698.

13 Jackson ME, Frost AS, Moghaddam B. Stimulation of prefrontal cortex at physiologically relevant frequencies inhibits dopamine release in the nucleus accumbens. *J Neurochem* 2001; **78**: 920–923.

14 Steinberg EE, Christoffel DJ, Deisseroth K, Malenka RC. Illuminating circuitry relevant to psychiatric disorders with optogenetics. *Curr Opin Neurobiol* 2015; **0**: 9–16.

15 Covington HE, Lobo MK, Maze I, Vialou V, Hyman JM, Zaman S *et al.* Antidepressant effect of optogenetic stimulation of the medial prefrontal cortex. *J Neurosci Off J Soc Neurosci* 2010; **30**: 16082–16090.

16 Robbins TW. Illuminating anhedonia. *Science* 2016; **351**: 24–25.

17 Maia TV, Frank MJ. From reinforcement learning models to psychiatric and neurological disorders. *Nat Neurosci* 2011; **14**: 154–162.

18 Gold JM, Waltz JA, Matveeva TM, Kasanova Z, Strauss GP, Herbener ES *et al.* Negative Symptoms and the Failure to Represent the Expected Reward Value of Actions: Behavioral and Computational Modeling Evidence. *Arch Gen Psychiatry* 2012; **69**: 129–138.

19 Groenewold NA, Opmeer EM, de Jonge P, Aleman A, Costafreda SG. Emotional valence modulates brain functional abnormalities in depression: Evidence from a meta-analysis of fMRI studies. *Neurosci Biobehav Rev* 2013; **37**: 152–163.

20 Zhang WN, Chang SH, Guo LY, Zhang KL, Wang J. The neural correlates of reward-related processing in major depressive disorder: A meta-analysis of functional magnetic resonance imaging studies. *J Affect Disord* 2013; **151**: 531–539.

21 Keren H, O’Callaghan G, Vidal-Ribas P, Buzzell GA, Brotman MA, Leibenluft E *et al.* Reward processing in depression: A conceptual and meta-analytic review across fMRI and EEG studies. *Am J Psychiatry* 2018; **175**: 1111–1120.

22 Arrondo G, Segarra N, Metastasio A, Ziauddeen H, Spencer J, Reinders NR *et al.* Reduction in ventral striatal activity when anticipating a reward in depression and schizophrenia: A replicated cross-diagnostic finding. *Front Psychol* 2015; **6**. doi:10.3389/fpsyg.2015.01280.

23 Bremner JD, Vythilingam M, Vermetten E, Charney DS. Effects of antidepressant treatment on neural correlates of emotional and neutral declarative verbal memory in depression. *J Affect Disord* 2007; **101**: 99–111.

24 Bürger C, Redlich R, Grotegerd D, Meinert S, Dohm K, Schneider I *et al.* Differential Abnormal Pattern of Anterior Cingulate Gyrus Activation in Unipolar and Bipolar Depression: an fMRI and Pattern Classification Approach. *Neuropsychopharmacology* 2017; **42**: 1399–1408.

25 Chantiluke K, Halari R, Simic M, Pariante CM, Papadopoulos A, Giampietro V *et al.* Fronto-Striato-Cerebellar Dysregulation in Adolescents with Depression During Motivated Attention. *Biol Psychiatry* 2012; **71**: 59–67.

26 Chase HW, Nusslock R, Almeida JRC, Forbes EE, LaBarbara EJ, Phillips ML. Dissociable patterns of abnormal frontal cortical activation during anticipation of an uncertain reward or loss in bipolar versus major depression. *Bipolar Disord* 2013; **15**: 839–854.

27 Dichter GS, Kozink RV, McClernon FJ, Smoski MJ. Remitted major depression is characterized by reward network hyperactivation during reward anticipation and hypoactivation during reward outcomes. *J Affect Disord* 2012; **136**: 1126–1134.

28 Elliott R, Rubinsztein JS, Sahakian BJ, Dolan RJ. The Neural Basis of Mood-Congruent Processing Biases in Depression. *Arch Gen Psychiatry* 2002; **59**: 597–604.

29 Engelmann JB, Berns GS, Dunlop BW. Hyper-responsivity to losses in the anterior insula during economic choice scales with depression severity. *Psychol Med* 2017; **47**: 2879–2891.

30 Fournier JC, Keener MT, Mullin BC, Hafeman DM, LaBarbara EJ, Stiffler RS *et al.* Heterogeneity of Amygdala Response in Major Depressive Disorder: The Impact of Lifetime Sub-Threshold Mania. *Psychol Med* 2013; **43**: 293–302.

31 Fu CHY, Williams SCR, Cleare AJ, Brammer MJ, Walsh ND, Kim J *et al.* Attenuation of the neural response to sad faces in major depression by antidepressant treatment: a prospective, event-related functional magnetic resonance imaging study. *Arch Gen Psychiatry* 2004; **61**: 877–889.

32 Fu CHY, Williams SCR, Brammer MJ, Suckling J, Kim J, Cleare AJ *et al.* Neural responses to happy facial expressions in major depression following antidepressant treatment. *Am J Psychiatry* 2007; **164**: 599–607.

33 Fu CHY, Williams SCR, Cleare AJ, Scott J, Mitterschiffthaler MT, Walsh ND *et al.* Neural responses to sad facial expressions in major depression following cognitive behavioral therapy. *Biol Psychiatry* 2008; **64**: 505–512.

34 Gotlib IH, Sivers H, Gabrieli JDE, Whitfield-Gabrieli S, Goldin P, Minor KL *et al.* Subgenual anterior cingulate activation to valenced emotional stimuli in major depression. *Neuroreport* 2005; **16**: 1731–1734.

35 Gradin VB, Pérez A, MacFarlane JA, Cavin I, Waiter G, Engelmann J *et al.* Abnormal brain responses to social fairness in depression: an fMRI study using the Ultimatum Game. *Psychol Med* 2015; **45**: 1241–1251.

36 Hall GBC, Milne AMB, MacQueen GM. An fMRI study of reward circuitry in patients with minimal or extensive history of major depression. *Eur Arch Psychiatry Clin Neurosci* 2014; **264**: 187–198.

37 Johnston BA, Tolomeo S, Gradin V, Christmas D, Matthews K, Steele JD. Failure of hippocampal deactivation during loss events in treatment-resistant depression. *Brain J Neurol* 2015; **138**: 2766–2776.

38 Keedwell PA, Andrew C, Williams SCR, Brammer MJ, Phillips ML. A double dissociation of ventromedial prefrontal cortical responses to sad and happy stimuli in depressed and healthy individuals. *Biol Psychiatry* 2005; **58**: 495–503.

39 Knutson B, Bhanji JP, Cooney RE, Atlas LY, Gotlib IH. Neural responses to monetary incentives in major depression. *Biol Psychiatry* 2008; **63**: 686–692.

40 Kumari V, Mitterschiffthaler MT, Teasdale JD, Malhi GS, Brown RG, Giampietro V *et al.* Neural abnormalities during cognitive generation of affect in treatment-resistant depression. *Biol Psychiatry* 2003; **54**: 777–791.

41 Laurent HK, Ablow JC. A Face a Mother Could Love: Depression-Related Maternal Neural Responses to Infant Emotion Faces. *Soc Neurosci* 2013; **8**: 228–239.

42 Liu W-H, Valton V, Wang L-Z, Zhu Y-H, Roiser JP. Association between habenula dysfunction and motivational symptoms in unmedicated major depressive disorder. *Soc Cogn Affect Neurosci* 2017; **12**: 1520–1533.

43 Murrough JW, Collins KA, Fields J, DeWilde KE, Phillips ML, Mathew SJ *et al.* Regulation of neural responses to emotion perception by ketamine in individuals with treatment-resistant major depressive disorder. *Transl Psychiatry* 2015; **5**: e509.

44 Pizzagalli DA, Holmes AJ, Dillon DG, Goetz EL, Birk JL, Bogdan R *et al.* Reduced caudate and nucleus accumbens response to rewards in unmedicated subjects with major depressive disorder. *Am J Psychiatry* 2009; **166**: 702–710.

45 Remijnse PL, Nielen MMA, van Balkom AJLM, Hendriks G-J, Hoogendijk WJ, Uylings HBM *et al.* Differential frontal-striatal and paralimbic activity during reversal learning in major depressive disorder and obsessive-compulsive disorder. *Psychol Med* 2009; **39**: 1503–1518.

46 Rizvi SJ, Salomons TV, Konarski JZ, Downar J, Giacobbe P, McIntyre RS *et al.* Neural response to emotional stimuli associated with successful antidepressant treatment and behavioral activation. *J Affect Disord* 2013; **151**: 573–581.

47 Rosenblau G, Sterzer P, Stoy M, Park S, Friedel E, Heinz A *et al.* Functional neuroanatomy of emotion processing in major depressive disorder is altered after successful antidepressant therapy. *J Psychopharmacol (Oxf)* 2012; **26**: 1424–1433.

48 Scheuerecker J, Meisenzahl EM, Koutsouleris N, Roesner M, Schöpf V, Linn J *et al.* Orbitofrontal volume reductions during emotion recognition in patients with major depression. *J Psychiatry Neurosci JPN* 2010; **35**: 311–320.

49 Schiller CE, Minkel J, Smoski MJ, Dichter GS. Remitted Major Depression is Characterized by Reduced Prefrontal Cortex Reactivity to Reward Loss. *J Affect Disord* 2013; **151**: 756–762.

50 Segarra N, Metastasio A, Ziauddeen H, Spencer J, Reinders NR, Dudas RB *et al.* Abnormal Frontostriatal Activity During Unexpected Reward Receipt in Depression and Schizophrenia: Relationship to Anhedonia. *Neuropsychopharmacology* 2016; **41**: 2001–2010.

51 Sharp C, Kim S, Herman L, Pane H, Reuter T, Strathearn L. Major depression in mothers predicts reduced ventral striatum activation in adolescent female offspring with and without depression. *J Abnorm Psychol* 2014; **123**: 298–309.

52 Smoski MJ, Rittenberg A, Dichter GS. Major depressive disorder is characterized by greater reward network activation to monetary than pleasant image rewards. *Psychiatry Res* 2011; **194**: 263–270.

53 Smoski MJ, Felder J, Bizzell J, Green SR, Ernst M, Lynch TR *et al.* FMRI of alterations in reward selection, anticipation, and feedback in major depressive disorder. *J Affect Disord* 2009; **118**: 69–78.

54 Surguladze SA, El-Hage W, Dalgleish T, Radua J, Gohier B, Phillips ML. Depression is associated with increased sensitivity to signals of disgust: a functional magnetic resonance imaging study. *J Psychiatr Res* 2010; **44**: 894–902.

55 Surguladze SA, Brammer MJ, Keedwell P, Giampietro V, Young AW, Travis MJ *et al.* A differential pattern of neural response toward sad versus happy facial expressions in major depressive disorder. *Biol Psychiatry* 2005; **57**: 201–209.

56 Townsend JD, Eberhart NK, Bookheimer SY, Eisenberger NI, Foland-Ross LC, Cook IA *et al.* fMRI activation in amygdala and orbitofrontal cortex in unmedicated subjects with major depressive disorder. *Psychiatry Res* 2010; **183**: 209–217.

57 Wang L, Krishnan KR, Steffens DC, Potter GG, Dolcos F, McCarthy G. Depressive state- and disease-related alterations in neural responses to affective and executive challenges in geriatric depression. *Am J Psychiatry* 2008; **165**: 863–871.

58 Young KD, Bodurka J, Drevets WC. Differential neural correlates of autobiographical memory recall in bipolar and unipolar depression. *Bipolar Disord* 2016; **18**: 571–582.

59 Zhang B, Li S, Zhuo C, Li M, Safron A, Genz A *et al.* Altered task-specific deactivation in the default mode network depends on valence in patients with major depressive disorder. *J Affect Disord* 2017; **207**: 377–383.

60 Zhong M, Wang X, Xiao J, Yi J, Zhu X, Liao J *et al.* Amygdala hyperactivation and prefrontal hypoactivation in subjects with cognitive vulnerability to depression. *Biol Psychol* 2011; **88**: 233–242.
